# Developing and validating an Interdisciplinary Teaching Readiness Scale (ITRS) for pre-service teachers in China

**DOI:** 10.1371/journal.pone.0315723

**Published:** 2024-12-31

**Authors:** Xin Wang, Lei Yuan, Shuang Li, Haoyuan Yu, Pan Tuo, Yongxiang Du, Baohui Zhang

**Affiliations:** 1 Faculty of Education, Shaanxi Normal University, Xi’an, China; 2 The Key Lab of Education Blockchain and Intelligent Technology, Ministry of Education, Guangxi Normal University, Guilin, China; 3 Faculty of Education, Guangxi Normal University, Guilin, China; 4 School of Music, Guangxi Normal University, Guilin, China; National University of Lesotho, LESOTHO

## Abstract

Assisting pre-service teachers in developing readiness for interdisciplinary teaching has been recognized as a crucial direction in teacher education in China. However, there is currently a lack of reliable instrument to measure the readiness. We developed and validated an Interdisciplinary Teaching Readiness Scale (ITRS) for pre-service teachers to fill the gap. We utilized literature review and interdisciplinary teaching competence framework to develop the initial item pool for the ITRS. Data were collected from 234 pre-service teachers at S Normal University (Sample A) for item analysis and exploratory factor analysis (EFA), followed by data collection from 543 pre-service teachers in China (Sample B) for confirmatory factor analysis(CFA), convergent validity, discriminant validity, and reliability testing. Item analysis on Sample A data using the critical ratio method revealed discriminative items, indicating no need for item deletion. PCA showed that the ITRS has a three-factor structure, explaining 77.282% of the total variance. CFA on Sample B demonstrated a good model fit (GMIN/DF = 4.189, RMSEA = 0.077, GFI = 0.852, AGFI = 0.821, NFI = 0.939, RFI = 0.932, CFI = 0.953, IFI = 0.953). Analysis of the AVE for each dimension indicated good convergent and discriminant validity. Reliability testing revealed a high overall Cronbach’s α coefficient of 0.972 for the ITRS, indicating good internal consistency. Ultimately, we confirmed that the ITRS consists of three factors(including interdisciplinary teaching knowledge structure readiness, interdisciplinary teaching skills readiness, interdisciplinary teaching attitudes readiness) and 24 items. In conclusion, the ITRS that has been developed shows great potential for promoting the professional development of pre-service teachers, evaluating the effectiveness of teacher education programs, and supporting the development of educational policy. The extensive utilization of this instrument will help to comprehensively assess the overall level of pre-service teachers’ readiness for interdisciplinary teaching and to promote the professional growth of pre-service teachers in China. Furthermore, the ITRS, through the implementation of culturally adaptable modifications, can offers invaluable instrumental support and insightful guidance to pre-service teacher education programs globally.

## Introduction

Interdisciplinary teaching, as a problem-solving oriented approach, integrates knowledge and methods from multiple disciplines [1,2]. It is increasingly valued globally in K-12 education as a means to cultivate creativity, critical thinking, collaboration, and communication skills [3]. This was explicitly reflected in the "2022 Edition of Compulsory Education Curriculum Scheme and Curriculum Standards" released by the Chinese Ministry of Education in April 2022, which stipulates that all courses in the compulsory education stage must ensure at least 10% of class hours are devoted to the design and implementation of interdisciplinary theme learning. This highlights China’s determination to comprehensively promote interdisciplinary teaching in the K-12 education.

Focusing on research into interdisciplinary teaching in China is significantly necessary. Firstly, interdisciplinary teaching far from being a common practice in schools globally, remaining rather experimental [3]. However, China demonstrates unprecedented policy implementation efforts and extensive coverage in promoting this teaching method, forming an innovative educational model that can be a reference for the international education community. Secondly, China has a vast number of pre-service teachers, with over 600,000 new teachers graduating from normal universities, comprehensive universities, or vocational colleges annually, and nearly two-thirds of them directly entering teaching positions [4]. Despite high expectations for new teachers to quickly demonstrate the same level of teaching professionalism as their experienced colleagues, insufficient teaching readiness is a common challenge faced by novice teachers worldwide [5]. Interdisciplinary teaching poses significant challenges to the restructuring of teaching content and methods, as well as to the teaching thinking and identity of pre-service teachers [3] which may result in issues such as low job satisfaction, anxiety, and turnover [6]. Consequently, to overcome this practical obstacle, it is imperative to guarantee comprehensive readiness for pre-service teachers prior to their commencement of duties. This approach will undoubtedly facilitate their success in their future pedagogical roles.

Despite the increasing research on pre-service teachers’ readiness and professional development in interdisciplinary teaching [7–9], 2020), there remains a lack of comprehensive assessment of pre-service teachers’ readiness in interdisciplinary teaching [10]. In order to ensure congruence between interdisciplinary teaching teacher preparation programs and the actual level of readiness among pre-service teachers, the development of a reliable and valid assessment instrument is of paramount importance [11]. Such an instrument would enable us to diagnose the efficacy of pre-service teacher readiness initiatives and ascertain the true extent of teachers’ readiness for interdisciplinary teaching.

In existing research on interdisciplinary teaching, survey questionnaires are the most commonly used instruments for assessing teachers’ interdisciplinary teaching readiness [12–14]. On one hand, some researchers have developed questionnaires to assess teachers’ readiness for interdisciplinary teaching from a systemic perspective, including the assessment of attitudes/emotional readiness, cognitive readiness, and behavioral readiness [15]. The attitudes/emotional readiness refers to the extent to which teachers evince interest and recognition of interdisciplinary teaching. This may be gauged, for instance, by the question, " I enjoy implementing STEM education approach in my lesson." Cognitive readiness refers to the knowledge structures and thinking patterns of teachers related to interdisciplinary teaching. This may be assessed by questions such as "I understand and master various knowledge contents and implementation methods of STEM education." Behavioral readiness refers to teachers’ interdisciplinary teaching practice performance. This includes whether they are able to implement effective interdisciplinary teaching in the classroom. This is demonstrated by questions such as, "I always analyze the existing personality characteristics and cognitive levels of students in order to carry out STEM education." [16]. On the other hand, researchers have elected to investigate teachers’ interdisciplinary teaching readiness in a specific domain with greater specificity [17,18]. For example, some researchers have highlighted the significant influence of teachers’ positive attitudes on fostering students’ interest and perceptions in STEM learning [19,20]. They developed a questionnaire designed to be used to assess teachers’ readiness for interdisciplinary (STEM) teaching attitudes, based on an instrument on perceptions of interdisciplinary curricula [21], which included three items assessing interdisciplinary teaching experience and 14 items assessing interdisciplinary teaching attitudes [22]. In addition, there are some researchers who pay particular attention to teachers’ overall view of interdisciplinary (STEM) teaching as one of the prerequisites for interdisciplinary teaching readiness [14,23]. Based on this, they have developed appropriate questionnaire to understand teachers’ perceptions and acceptance of interdisciplinary teaching. For example, Kanmaz developed a questionnaire that emphasized the importance of teachers’ perspectives on the implementation of interdisciplinary teaching, the questionnaire containing three dimensions: the benefits of the interdisciplinary approach, the interdisciplinary teaching practices and the place of the interdisciplinary approach in the curriculum [23].

Although these instruments are widely used and effective, the existing literature highlights a limitation: It is not uncommon for many research developed questionnaires to be lacking in a solid theoretical framework at the early stages of their design, the validity and reliability of such questionnaires are not always subjected to sufficient scrutiny [16,24]. This highlights the urgent need to develop scientifical assessment instruments. In contrast to questionnaires, scales require a more comprehensive structural design with clear theoretical distinctions between dimensions and strong internal consistency [25,26]. This leads to the first research question of this study, which is to identify the dimensions of the Interdisciplinary Teaching Readiness Scale (ITRS). Moreover, unlike questionnaires, scales must undergo a series of validation procedures to ensure their reliability and validity prior to their formal use [27]. In educational research, methods such as exploratory factor analysis (EFA), confirmatory factor analysis (CFA), and alpha reliability are commonly used to assess the validity and reliability of scales [28–30]. This raises our second research question: Is it possible to construct and validate the ITRS model by applying EFA and CFA?

In consideration of the aforementioned context, the research questions of this study are as follows:

1. What are the constituent dimensions of the ITRS?
2. Is it possible to construct and validate the ITRS model by applying EFA and CFA?

In conclusion, there is an urgent need for a scientifically reliable assessment scale to investigate the preparedness of pre-service teachers in China for interdisciplinary teaching. This has significant implications for driving transformative reforms in Chinese teacher education and furnishes valuable insights for pre-service teacher education on a global scale.

## Conceptual framework

The core of this study is to develop and validate a scale for ITRS for Chinese pre-service teachers. Through literature review, we found no scientifically reliable scale for assessing pre-service teachers’ interdisciplinary teaching readiness. Drawing upon the interdisciplinary teaching competence framework, this study emphasizes its significant role in assessing pre-service teachers’ interdisciplinary teaching readiness.

### Teaching readiness and measurement of teaching readiness

Teaching readiness is broadly defined as the state of faculty preparation [31]. It refers to the level of ability and willingness exhibited by teachers in their teaching duties [32], and is typically used to estimate teachers’ competence in certain aspects or even the entirety of their work, such as readiness for blended learning [11]. Generally, the level of teaching readiness reflects teachers’ predictions of their own levels of teaching knowledge and skills, largely depending on their self-efficacy in this regard [33].

As seen in some reports related to teacher job descriptions, the responsibilities and obligations of teachers related to teaching are widely referred to as knowledge, skills, values, or attitudes related to teaching, and are represented as "teacher standards" or "teacher competencies or competences" [34]. Therefore, we believe that the measurement basis of teaching readiness generally falls into two categories:

The first category is based on "teacher professional standards" [35]. The formulation and implementation of teacher professional standards standardize teacher training work and are the professional qualities and basic norms that teachers need to meet teaching requirements. This type of survey is commonly used to assess the overall situation of pre-service teachers’ readiness for teaching.

The second category is based on "teacher’s teaching competence ". Teaching competence refers to the comprehensive personal characteristics, knowledge, skills, and attitudes that teachers exhibit in various teaching environments, and can serve as a guide for teachers’ self-assessment and improvement of teaching [36]. If pre-service teachers can acquire a clear and measurable set of teaching competence during teacher readiness, it is very likely to clarify and improve their teaching readiness level [37]. Therefore, teaching competence can "serve as a marker or reference point for judgments about the preparedness of beginning teachers" [38,39]. The connection between teaching competence and teaching readiness highlights the importance of clear, measurable standards for assessing teacher readiness. One effective way to evaluate teaching competence is through self-report assessments, which allow teachers to reflect on and evaluate their own skills, knowledge, and attitudes by completing scale items. This method not only provides insights into a teacher’s current level of readiness but also enables teacher educators and teachers themselves to formulate targeted improvement plans for the next steps in their professional development.

### Interdisciplinary teaching readiness

In existing research, surveys on interdisciplinary teaching readiness have led to the development of questionnaires. For example, Sinelnikov & Zharkovskaya developed a 14-item questionnaire surveying 258 teachers in Russia about their understanding of interdisciplinary teaching and its implementation [14]. Fidalgo-Neto et al. created a questionnaire covering teachers’ understanding of interdisciplinary themes, training background, views on interdisciplinary teaching importance, and school implementation [12]. Despite these developments, existing instrument lack theoretical framework guidance and factor analysis validation [40], highlighting the need for improved measurement instrument with enhanced reliability and systematicity.

### Interdisciplinary teaching competence framework

The interdisciplinary teaching competence framework describes the knowledge, abilities and attitudes required by teachers in interdisciplinary teaching and serves as a reference point for judging the readiness of pre-service teachers [38]. A review of existing research was conducted on interdisciplinary teaching competence. Given that STEM education represents a typical form of interdisciplinary teaching, the review also encompassed STEM teacher competence frameworks. Educational researchers have developed many models of teachers’ interdisciplinary teaching competence [17,41–46].

Existing models illustrate that interdisciplinary teaching competence encompasses key elements such as multidisciplinary knowledge, instructional implementation, and teaching beliefs, and provide preliminary guidance for teacher education. However, they face limitations. Firstly, existing frameworks for interdisciplinary teaching competence are closely related to those for STEM teacher competence. As a broader concept than STEM education, interdisciplinary teaching involves a greater variety of disciplinary integrations and focuses more on fostering students’ broad literacy and cross-disciplinary thinking skills [47]. The direct transplantation or substitution of interdisciplinary teaching competency frameworks with STEM teacher competency frameworks reveals a lack of necessary adaptability and flexibility in the competency frameworks [48].Secondly, the structural exploration of current interdisciplinary teaching competence models primarily adheres to logical conceptual explanations or relies heavily on reasoning without corresponding empirical research support [41,49]. Finally, while in-service teachers can usually develop flexible interdisciplinary teaching implementation skills over time and through extensive experience, pre-service teachers have limited opportunities for practice [50]. Therefore, when designing a competency framework for interdisciplinary teaching, emphasis should be placed not only on practical teaching skills but also on the importance of knowledge relevant to interdisciplinary teaching to ensure that pre-service teachers can develop adaptable interdisciplinary teaching skills based on a solid understanding of relevant concepts. Under these circumstances, the development of a framework that takes into account the unique characteristics of both interdisciplinary teaching and pre-service teachers becomes imperative.

Despite the aforementioned shortcomings of existing frameworks, they have been validated through practical application and, in particular, provide invaluable insights into the detailed aspects of teachers’ interdisciplinary teaching implementation competencies [3,51]. Therefore, in developing a framework tailored to pre-service teachers’ interdisciplinary teaching implementation competencies, we have taken advantage of the strengths of established frameworks. These include their careful descriptions of specific competencies such as lesson planning, implementation, and assessment [52]. This will ensure that the interdisciplinary teaching competency framework we have constructed will better serve the development of preservice teachers and lay the groundwork for their future engagement in interdisciplinary teaching.

To develop a generic competency framework of interdisciplinary teaching competencies for pre-service teachers, we conducted a review of both Chinese and English literature related to interdisciplinary teaching competence and STEM teacher competence. The two major frameworks were selected as the main references for the study due to their rigorous theoretical constructs, broad influence, and in-depth analysis of STEM education practices in China and internationally. Firstly, we adopted the "STEM Teacher Competency Standards (Trial)" released by the Chinese Academy of Educational Sciences in 2018. This standard outlines five key competency elements, including awareness of STEM education values, foundational knowledge in STEM subjects, interdisciplinary understanding and practical application in STEM, development and integration of STEM curriculum, and implementation and evaluation of STEM teaching. Secondly, we referenced "STEM EDUCATION FRAMEWORK" published by The New York Academy of Sciences. This report emphasizes that high-quality STEM education consists of three pillars: Core Competencies, Instructional Design, and Implementation.

In addition, the onion ring model provides a hierarchical perspective on teacher competencies that helps us to divide interdisciplinary teaching competencies into three interrelated and deeper dimensions of knowledge, skills, and attitudes, thus providing a more specific and hierarchical way to design an interdisciplinary teaching competency framework [53,54]. Therefore, we referenced the "Layers of the Onion Ring Model for Teachers’ Professional Development" [55] and based on the two frameworks mentioned above, we mapped the interdisciplinary teaching competencies of pre-service teachers on three dimensions: interdisciplinary teaching knowledge structure, interdisciplinary teaching skills, and interdisciplinary teaching attitudes.

Interdisciplinary teaching knowledge Structure refers to the ability of teachers to not only possess a solid foundation in individual subject areas but also to integrate knowledge from other disciplines and apply it in teaching activities. According to the Pedagogical Content Knowledge (PCK) theory, pre-service teachers’ interdisciplinary teaching knowledge can be divided into three components: multidisciplinary knowledge, interdisciplinary pedagogical knowledge, and the ability to effectively integrate interdisciplinary content with pedagogical methods in practical teaching contexts [56]. Interdisciplinary teaching skills refer to teachers’ abilities to design, implement, and evaluate interdisciplinary teaching tasks. This includes designing interdisciplinary teaching objectives, themes, content, and modes [57], implementing teaching strategies and guiding the learning process [41], and evaluating students’ interdisciplinary learning outcomes using diverse assessment methods [58]. Interdisciplinary teaching attitude reflects teachers’ willingness to engage in interdisciplinary teaching and includes their attitudes, values, beliefs, and judgments regarding interdisciplinary methods. It comprises interdisciplinary teaching beliefs, professional development willingness, and teaching attitudes.

## Methodology

In this section, we discuss the process of scale development, the research procedure, the participants of the study, and the data analysis methods used.

### Initial item development

We utilized literature review and expert consultation to generate items for the ITRS [59]. From literature related to ITRS indicators, we constructed a survey framework based on a generic framework of interdisciplinary teaching competence. Three indicators were identified: readiness in interdisciplinary teaching structure knowledge, skills, and attitudes, comprising 9 secondary dimensions and 24 tertiary items. The details are presented in Table 1.

**Table 1:** Survey framework for pre-service teachers’ interdisciplinary teaching readiness.

	Primary Indicators	Secondary Dimensions	Tertiary Items	Reference
Pre-Service Teachers’ Interdisciplinary Teaching Readiness	Interdisciplinary Teaching Knowledge Structure Readiness	Interdisciplinary Knowledge	Interdisciplinary Conceptual Understanding, Subject Matter Knowledge Reservoir, Interdisciplinary Logical Connections	[60,61]
		Pedagogical Knowledge in Interdisciplinary Teaching	Various teaching methods applicable to interdisciplinary teaching (such as PBL, engineering design-based teaching, 5E teaching method, etc.)	
		Pedagogical Content Knowledge in Interdisciplinary Teaching	Applying teaching methods in specific teaching contexts to facilitate interdisciplinary teaching and promote understanding of knowledge	
	Interdisciplinary Teaching Skills Readiness	Interdisciplinary Teaching Design skills	Goal design, theme design, content design, and mode selection	STEM Teacher Competency Standards (Trial);STEM EDUCATION FRAMEWORK
		Interdisciplinary Teaching Implementation Skills	Creating teaching scenarios, organizing classroom activities, using teaching strategies, and guiding the learning process	
		Interdisciplinary Teaching Evaluation Skills	The multidimensionality of evaluation content, the diversity of evaluation methods, and the diversity of evaluation subjects	
	Interdisciplinary Teaching Attitudes Readiness	Interdisciplinary Teaching Beliefs	Value understanding, essence comprehension	STEM Teacher Competency Standards (Trial);InTASC Model Core Teaching Standards
		Professional Development Willingness	Participation in training, resource seeking, teaching adjustment	
		Interdisciplinary Teaching Attitude	Identification, interest, expectation	

On the basis of the survey framework for pre-service teachers’ interdisciplinary teaching readiness, we developed the initial item pool for the ITRS, consisting of 25 items. Example items include: "I am familiar with teaching methods such as problem-based learning, project-based learning, engineering design-based teaching, and the 5E teaching model," "I can identify appropriate learning topics based on the difficulty and complexity of real-life situations from students’ lives," "I am very eager to engage in interdisciplinary teaching in my future teaching work." We then translated these items into Chinese and invited expert judges to participate in the content validity assessment of the ITRS to ensure that the items reflected the content we intended to measure [62]. Content validity refers to the degree to which a scale actually measures what it is intended to measure. This is an important indicator of the quality of the scale, and it is therefore essential to carry out this assessment step [63]. In order to achieve this objective, a panel comprising three university professors has been convened, including the fields of educational technology (n = 2) and teacher education (n = 1). Collectively, these three experts have a profound understanding of interdisciplinary teaching, STEM education. Each of them reviewed all 25 original items and discussed their representativeness for the construct.

In order to provide evidence of content validity, scale developers typically calculate a content validity index (CVI) [63,64], which consists of an item-level CVI (I-CVI) and a scale-level CVI (S-CVI) [65]. These indices are calculated based on the ratings provided by experts. Specifically, the researcher invites experts to rate the relevance or representativeness of each entry in the scale to its corresponding content dimension on a scale ranging from 1 (not relevant) to 4 (very relevant). Additionally, the researcher encourages experts to make suggestions for needed additions, deletions, or adjustments to the entries [66]. The I-CVI is calculated as the ratio of the number of experts who rated 3 or 4 for each entry to the total number of experts who participated in the review. In the event that the number of experts does not exceed 5, the I-CVI should ideally reach 1.00, indicating unanimous agreement amongst experts that the entry is appropriately related to the concept under examination [67,68]. The S-CVI is divided into two categories: S-CVI/UA (universal agreement) and S-CVI/Ave. S-CVI/UA is calculated by determining the proportion of all entries that receive a rating of 3 or 4 from the experts. When S-CVI/UA is not less than 0.8, it indicates good content validity of the scale [69]. There are three methods of calculating S-CVI/Ave: the mean of the I-CVI of all the entries of the scale; the mean of the proportion of the entries that received a rating of 3 or 4 from each expert; the 3 or 4 rating occurrences divided by the total number of ratings [70]. It is generally accepted that S-CVI/Ave should be above 0.90 [71].

The results of our calculations indicated that I-CVI, S-CVI/UA and S-CVI/Ave were all equal to 1.00. These figures provide compelling evidence that the three experts recognize the content validity of the ITRS to a high degree. In light of the textual suggestions proffered by the experts, minor adjustments were made to the presentation of certain questions with a view to further optimizing the precision and clarity of the presentation of each question. However, it became evident that, despite these experts’ considerable experience in their respective fields, there may be inherent limitations in relying on a limited number of experts to assess content validity. Consequently, the initial questionnaire was also pretested when it was distributed to collect sample A. In particular, the understanding of and feedback on the questionnaire questions provided by the initial participants were collected in order to test the actual validity of the questions and make any necessary adjustments to the initial test items. Furthermore, data analyses, including exploratory and validation factor analysis, were conducted to assess the rationality of the questionnaire structure during the subsequent scale development process.

ITRS is managed through Wenjuanxing (You can see the live instrument at https://www.wjx.cn/vm/rXVGkr1.aspx#), and it consists of two parts: basic information and interdisciplinary teaching readiness, totaling 36 items. The first part, basic information, includes 11 questions covering gender, grade, major category, type of institution, learning experience, training experience, teaching (internship) experience. The second part, interdisciplinary teaching readiness, comprises 25 items aimed at assessing pre-service teachers’ readiness for interdisciplinary teaching. It primarily investigates three dimensions: interdisciplinary teaching knowledge, interdisciplinary teaching skills, and interdisciplinary teaching attitudes. Except for the 11 items in the basic information section, participants rate the accuracy of each statement on a scale of 1 to 5, where "1" indicates strongly disagree and "5" indicates strongly agree. A higher total score on the scale indicates a higher level of readiness for interdisciplinary teaching.

### Procedure

This study first utilized an initial scale to measure the initial participants from Shaanxi Normal University. The recruitment period for Sample A, began on January 5, 2024, and concluded on February 15, 2024. The data from sample A were subjected to item analysis and EFA. Based on the analysis results, adjustments were made to factors and items, resulting in the formation of the main survey scale for interdisciplinary teaching readiness. Subsequently, a formal scale was distributed nationwide in China. The collection of data for Sample B commenced on March 20, 2024, and ended on May 30, 2024, yielding a total of 543 valid responses. CFA, convergent validity, discriminant validity, and reliability tests were conducted using the data from sample B.

### Participants

Ethical approval was obtained from the Ethics Committee of Guangxi Normal University. Written informed consent was confirmed by all participants. Sample A comes from Shaanxi Normal University, which is a key university under the "211 Project" directly managed by the Ministry of Education. It has extensive social influence in the field of teacher education [72]. The random sampling method is a straightforward approach to forming a sample group and is a viable data collection strategy that can be employed in research. This method ensures that all members of a given population have an equal opportunity for selection [73]. We adopted a random sampling method to conduct an online scale survey among students from different majors and grades. In the end, we obtained a total of 234 valid questionnaires.

Sample B employed a stratified random sampling method. The application of stratified random sampling ensures that each subgroup of the population is adequately represented in the sample, thereby providing a more comprehensive overall coverage [74]. Firstly, we divided China into four primary sampling units: Eastern, Central, Western, and Northeastern regions, based on the division of economic regions by the National Bureau of Statistics of China. Then, within each unit, two teacher education colleges or comprehensive universities were selected as secondary sampling units. Finally, according to the principle of random sampling, we distributed and collected at least 50 questionnaires in each selected institution, resulting in a total of 543 collected questionnaires. Table 2 provides detailed demographic data for each participant in Sample A and Sample B.

**Table 2:** Demographic characteristics of the samples.

Characteristics	Sample A (n = 234)	Sample B (n = 543)
**Gender**		
Female	192 (82.1%)	445(82%)
Male	42(63%)	98(18%)
**Grade**		
Freshman	66(28.2%)	179(33%)
Sophomore	59(25.2%)	104(19.2%)
Junior	59(25.2%)	214(39.4%)
Senior	50(21.4%)	46(8.5)
**Economic regions**		
Eastern	0	132(24.3%)
Western	234(100%)	185(34.1%)
Central	0	103(19.0%)
Northeastern	0	123(22.7%)
**Major Category**		
Humanities and Social Sciences	127(54.3%)	381 (70.2%)
Science and Engineering	107(45.7%)	162 (29.8%)
**Educational experience**		
Yes	28 (12%)	63(11.6%)
No	206(88%)	480(88.4%)
**Training experience**		
Yes	20(8.5%)	50(9.2%)
No	214(91.5%)	493(90.8%)
**Teaching experience**		
Yes	10(4.3%)	33(6.1%)
No	224(95.7%)	510(93.9%)

### Data analysis process

Sample A underwent item analysis and exploratory factor analysis (EFA), while Sample B was used for confirmatory factor analysis (CFA), convergent validity, discriminant validity, and reliability testing. In the item analysis of Sample A, we utilized the critical ratio (CR) to evaluate item appropriateness. For EFA, principal component analysis (PCA) was employed to extract factors. We assessed the suitability of the data for EFA using the KMO measure and Bartlett’s test of sphericity, followed by factor rotation and removal of inadequate items based on factor loadings. CFA of Sample B was conducted using the maximum likelihood method in AMOS 26.0 software, preceded by a test of normality. Convergent validity and discriminant validity were analyzed, focusing on correlations among measurement items within factors and between factors, respectively [75]. Reliability testing employed Cronbach’s alpha coefficient. These methods systematically examined the validity and reliability of the scale.

## Results

### Item analysis

We conducted item analysis on Sample A data using the critical ratio method, dividing total scores into high and low score groups [28]. Critical values for high and low score groups were set at 91 and 74 points, respectively, representing the top 27% and bottom 27% of scores. Table 3 illustrates the difference test between these groups for each item. Results showed significant differences between high and low score groups for all items. Therefore, all 25 items of the ITRS for predicting the interdisciplinary teaching readiness of pre-service teachers in China were deemed reasonable, with no need for item deletion.

**Table 3:** Independent samples t-test results for high-low group comparison.

Items	Mean ± Standard Deviation		t	p
	High Score Group (N = 69)	Low Score Group (N = 69)		
KS1	3.03±0.954	1.84±0.868	7.651	0.000
KS2	3.28±0.968	1.86±0.713	9.812	0.000
KS3	3.46±0.901	2.23±0.91	7.993	0.000
KS4	3.61±0.808	2.03±0.766	11.780	0.000
KS5	3.30±0.896	1.72±0.684	11.643	0.000
KS6	3.36±0.857	1.78±0.704	11.829	0.000
TS1	3.75±0.793	2.12±0.676	13.051	0.000
TS2	3.94±0.662	2.28±0.82	13.136	0.000
TS3	3.99±0.581	2.19±0.713	16.229	0.000
TS4	3.90±0.667	2.22±0.764	13.761	0.000
TS5	4.00±0.569	2.32±0.675	15.820	0.000
TS6	3.93±0.714	2.22±0.82	13.066	0.000
TS7	4.09±0.612	2.17±0.593	18.646	0.000
TS8	4.09±0.588	2.28±0.684	16.695	0.000
TS9	4.17±0.617	2.48±0.779	14.176	0.000
TS10	4.07±0.671	2.45±0.758	13.317	0.000
TS11	4.20±0.584	2.38±0.769	15.714	0.000
TA1	4.42±0.604	3.12±1.022	9.124	0.000
TA2	4.43±0.606	2.96±0.93	11.060	0.000
TA3	4.55±0.582	3.07±1.005	10.574	0.000
TA4	4.51±0.609	3.03±0.939	10.971	0.000
TA5	4.55±0.501	3.1±0.957	11.144	0.000
TA6	4.55±0.557	3.25±1.035	9.221	0.000
TA7	4.42±0.604	3.06±1.042	9.399	0.000
TA8	4.52±0.633	3.07±1.034	9.934	0.000

### Exploratory Factor Analysis (EFA)

EFA is a method used to explore the underlying structure of scale data and identify patterns. Sample A was utilized for EFA to examine the structure of the Pre-Service Teachers ITRS in China. The KMO coefficient is 0.943 (>0.6), and the Bartlett test result is significant (χ2 = 6753.08, df = 300, p < 0.001), indicating the suitability of the ITRS for factor analysis. We conducted a PCA with varimax rotation on the 25 items to explore the underlying structure of the ITRS.

The PCA results show a three-factor structure for the ITRS, explaining 77.282% of the total variance. Factor 1 comprises 6 items (32.49% variance), labeled "Knowledge Structure" (KS). Factor 2 consists of 11 items (25.783% variance), named "Teaching Skills" (TS). Factor 3 includes 8 items (19.01% variance), labeled "Teaching Attitude" (TA). Following the principle of deleting items with cross-loadings or low factor loadings, we removed item TS1 from the ITRS and marked it with "—", resulting in 24 remaining items. Table 4 displays the results of the PCA for the ITRS.

**Table 4:** Results of the PCA with varimax rotation.

Items	Knowledge Structure	Teaching Skills	Teaching Attitude
KS1	0.821		
KS2	0.817		
KS3	0.711		
KS4	0.690		
KS5	0.852		
KS6	0.821		
TS2		0.821	
TS3		0.804	
TS4		0.847	
TS5		0.852	
TS6		0.767	
TS7		0.823	
TS8		0.837	
TS9		0.807	
TS10		0.760	
TS11		0.804	
TA1			0.720
TA2			0.735
TA3			0.829
TA4			0.890
TA5			0.844
TA6			0.831
TA7			0.853
TA8			0.835

Variance explained: Knowledge Structure = 32.49%; Teaching Skills = 25.783%; t Teaching Attitude = 19.01%.

### Confirmatory Factor Analysis (CFA)

We conducted CFA using the maximum likelihood method in AMOS 26.0 to analyze the data. CFA is used to examine the relationship between a set of measurement items and the latent factors that explain these measurement items. To ensure the scientific validity of the results, it is recommended to avoid using the same sample for both confirmatory and exploratory factor analyses. Therefore, we utilized sample B data for the CFA.

Prior to CFA, the normality of the data from sample B was assessed. Table 5 displays the normality test results, showing skewness<3 and kurtosis<8 for all items, indicating a normal distribution. Thus, the maximum likelihood method was suitable for model validation.

**Table 5:** Normality test results.

Items	skew	kurtosis	Items	skew	kurtosis
KS1	0.143	-0.09	TS8	-0.143	0.323
KS2	0.033	-0.134	TS9	-0.144	0.23
KS3	-0.148	0.144	TS10	-0.132	0.252
KS4	-0.119	0.129	TS11	-0.162	0.332
KS5	0.005	0.012	TA1	-0.239	-0.233
KS6	-0.03	0.007	TA2	-0.213	-0.145
TS2	-0.187	0.297	TA3	-0.404	-0.126
TS3	-0.194	0.326	TA4	-0.166	-0.397
TS4	-0.185	0.287	TA5	-0.333	0.034
TS5	-0.125	0.39	TA6	-0.343	-0.034
TS6	-0.077	0.337	TA7	-0.214	-0.161
TS7	-0.045	0.243	TA8	-0.266	-0.11

After confirming the normality of the data, we used CFA to examine the structural validity of the scale. The standards for testing the factor structure are mainly reflected in the fit indices of the observed measurement model, as shown in Table 6. It is worth noting that GFI or AGFI scores between 0.80 and 0.89 can be interpreted as reasonable fit [76]. Considering the sample size and index situation, we consider GFI > 0.85 as acceptable.

**Table 6:** CFA fit indices values.

Indexes	Acceptable Values	Initial	Interpretation	Revised	Interpretation
GMIN/DF	<5	4.878	Acceptable	4.189	Acceptable
RMSEA	<0.08	0.085	-	0.077	Acceptable
GFI	>0.85	0.826	-	0.852	Acceptable
AGFI	>0.8	0.790	-	0.821	Acceptable
NFI	>0.9	0.929	Acceptable	0.939	Acceptable
RFI	>0.9	0.921	Acceptable	0.932	Acceptable
CFI	>0.9	0.943	Acceptable	0.953	Acceptable
IFI	>0.9	0.943	Acceptable	0.953	Acceptable

**Fig 1:**
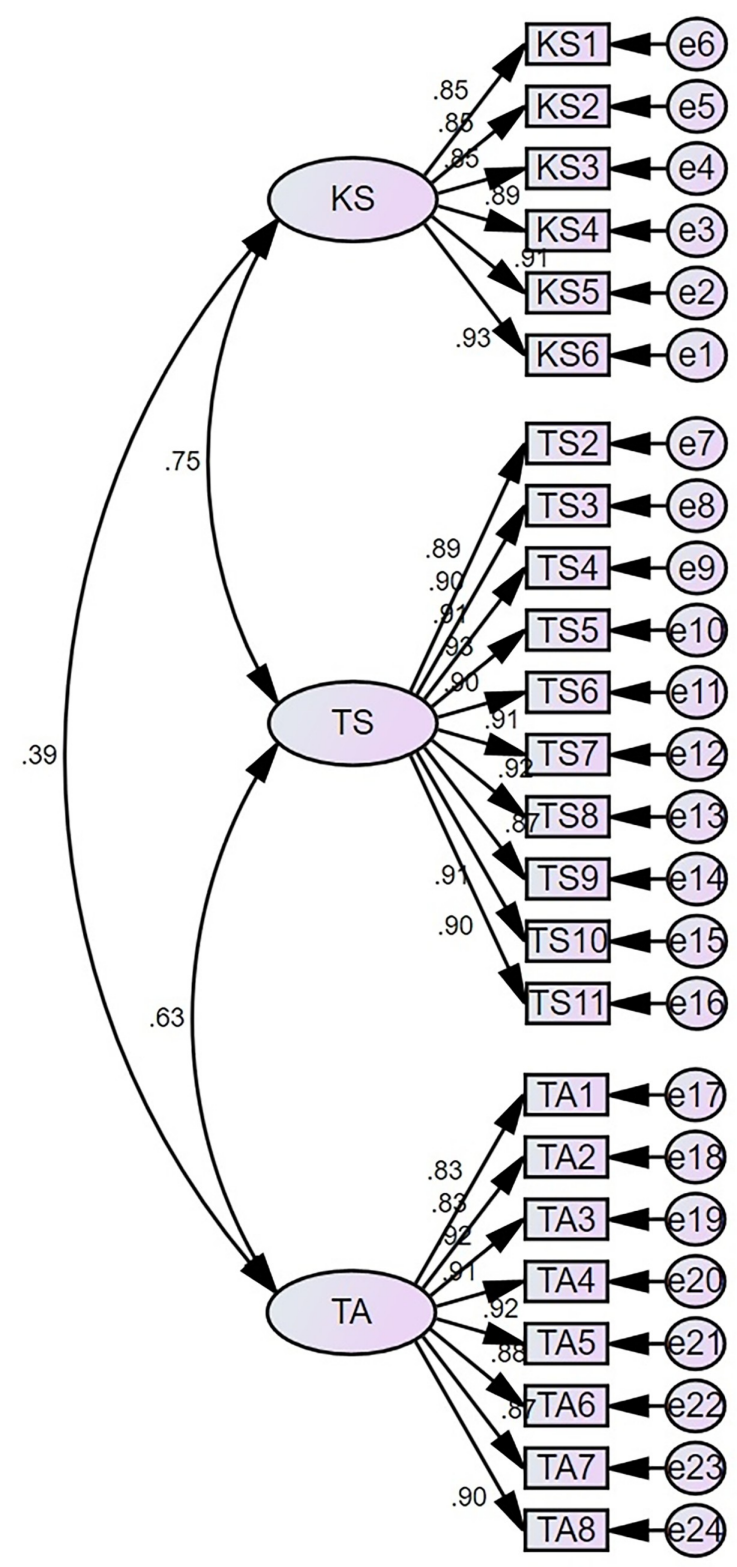
The initial CFA results.

First, we plotted three latent variables: Knowledge Structure (KS), Teaching Skills (TS), Teaching Attitude (TA), 24 observed variables, and 24 residual terms on the AMOS panel. Then, we constructed the relationships between the latent variables. Sample B data were imported into the AMOS 26.0 software for statistical analysis. Fig 1 represents the initial model results of CFA, and Table 6 displays the fit indices of the initial model. The results show that GMIN/DF = 4.878, RMSEA = 0.085, GFI = 0.826, AGFI = 0.790, NFI = 0.929, RFI = 0.921, CFI = 0.943, IFI = 0.943. Among them, RMSEA, GFI, and AGFI do not meet the criteria for fit indices, indicating that the model needs further refinement.

**Fig 2:**
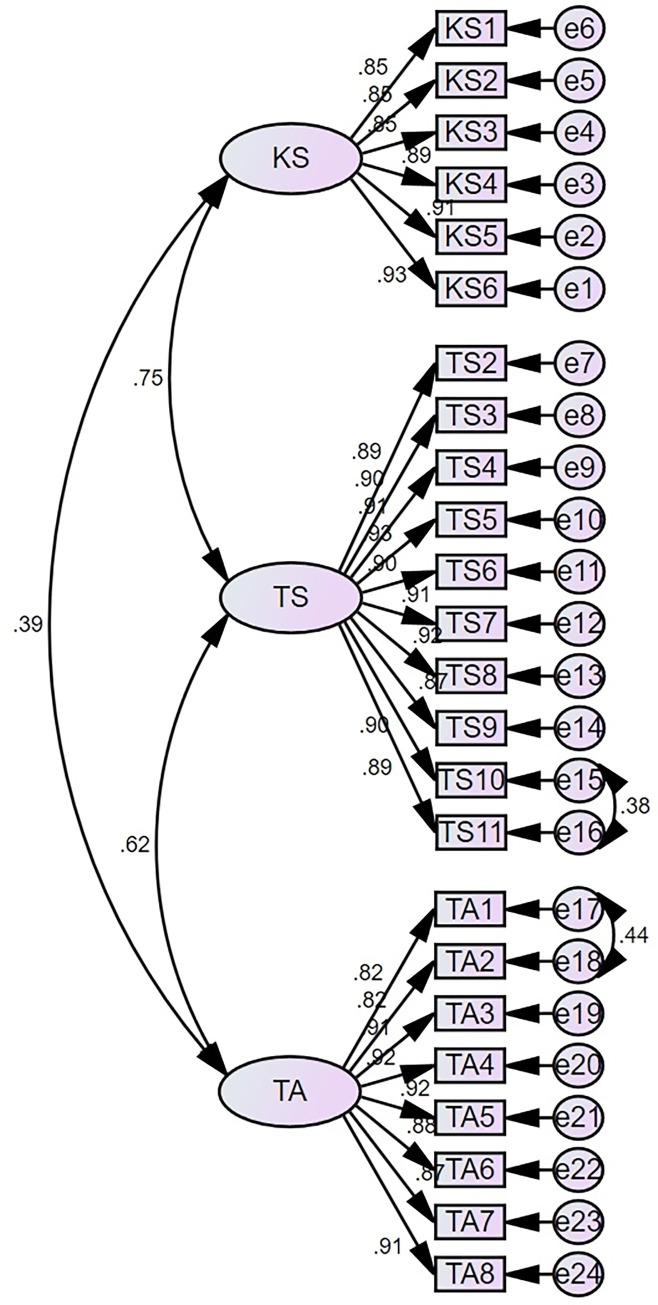
The revised CFA results.

Based on the initial model, we made adjustments by referring to the Modification Indices (MI) displayed in the output results of AMOS 26.0, indicating the modification needed between the error terms of each measurement item. Correlation paths were established sequentially between error variables e17<—>e18 (100.848) and e15<—>e16 (68.381). Fig 2 displays the results of the modified CFA. Table 6 shows the fit indices of the modified model. According to the results, the fit indices that did not initially meet the criteria, namely RMSEA, GFI, and AGFI, have now reached the standards for model fit.

### Convergent validity and discriminant validity

We further examined the convergent and discriminant validity of the scale. Convergent validity refers to the correlation among items within the same factor. Based on the output results from AMOS, we calculated the AVE and CR values for the three dimensions. Table 7 presents the results of the convergent validity test. The results indicate that the factor loadings of the measurement items corresponding to the variables Knowledge Structure (KS), Teaching Skills (TS), and Teaching Attitude (TA) are all greater than 0.6, suggesting good representativeness of the respective latent variables. Additionally, both the AVE and CR values for each latent variable meet the standards (AVE > 0.5, CR > 0.8), indicating satisfactory convergent validity of the scale.

**Table 7:** Convergent validity test results.

Path			Standard Estimate	AVE	CR
KS1	<—	Knowledge Structure	0.93	0.776	0.954
KS2	<—	Knowledge Structure	0.908		
KS3	<—	Knowledge Structure	0.889		
KS4	<—	Knowledge Structure	0.853		
KS5	<—	Knowledge Structure	0.853		
KS6	<—	Knowledge Structure	0.849		
TS2	<—	Teaching Skills	0.891	0.814	0.9777
TS3	<—	Teaching Skills	0.905		
TS4	<—	Teaching Skills	0.909		
TS5	<—	Teaching Skills	0.927		
TS6	<—	Teaching Skills	0.897		
TS7	<—	Teaching Skills	0.913		
TS8	<—	Teaching Skills	0.917		
TS9	<—	Teaching Skills	0.869		
TS10	<—	Teaching Skills	0.898		
TS11	<—	Teaching Skills	0.895		
TA1	<—	Teaching Attitude	0.816	0.7755	0.965
TA2	<—	Teaching Attitude	0.818		
TA3	<—	Teaching Attitude	0.915		
TA4	<—	Teaching Attitude	0.915		
TA5	<—	Teaching Attitude	0.917		
TA6	<—	Teaching Attitude	0.879		
TA7	<—	Teaching Attitude	0.873		
TA8	<—	Teaching Attitude	0.905		

Divergent validity refers to the distinctiveness of the relationships between the factors of ITRS. We compare the standardized coefficients between each latent variable and the square root of the AVE for each latent variable to test the divergent validity of the scale. Table 8 displays the results of the test for divergent validity.

**Table 8:** Discrimination validity test results.

	Knowledge Structure	Teaching Skills	Teaching Attitude
**Knowledge Structure**	0.776		
**Teaching Skills**	0.749***	0.814	
**Teaching Attitude**	0.385***	0.617***	0.776
**Square root of the AVE**	0.881	0.902	0.881

Note

*** indicates significance at the 0.001 level; the diagonal represents the AVE; values in the lower triangle represent the correlation coefficients between latent variables.

The results indicate that there are significant correlations (p < 0.01) among Knowledge Structure (KS), Teaching Skills (TS), and Teaching Attitude (TA). Additionally, the correlation coefficients are all smaller than the square root of their corresponding AVE, indicating that the latent variables have certain correlations while also maintaining distinctiveness from each other. This suggests that the discriminant validity of the scale data is ideal.

### Reliability analysis

Reliability refers to the consistency and stability within the scale, also known as the reliability of the questionnaire. We used the commonly used α reliability, calculated by Cronbach’s alpha coefficient, to assess the reliability of the scale. Table 9 shows the test results. The overall Cronbach’s α coefficient is 0.972, and the Cronbach’s α coefficients for Knowledge Structure, Teaching Skills, and Teaching Attitude are 0.954, 0.978, and 0.966, respectively, all exceeding the standard of 0.9. This indicates that the reliability of the scale data is good.

**Table 9:** Cronbach’s α reliability test results.

Dimension	Knowledge Structure	Teaching Skills	Teaching Attitude	Overall
Cronbach’s α coefficient	0.954	0.978	0.966	0.972

## Discussion

The aim of this study is to develop and validate a ITRS for measuring the interdisciplinary teaching readiness of pre-service teachers in China. Overall, the results of PCA provide evidence for a clear and meaningful three-factor structure of the ITRS. Further CFA demonstrates excellent structural validity, while the supplementary tests of convergent and discriminant validity complement this, indicating satisfactory measurement performance of the ITRS. This section discusses the following issues: (1) Dimensionality of the construct; (2) the reliability and validity of the scale.(3) Theoretical and practical implications.

### Dimensionality of the construct

We reviewed the conceptual framework of interdisciplinary teaching readiness and developed a scale suitable for pre-service teachers based on a general framework of interdisciplinary teaching competencies. Through principal component analysis, we revealed a scale consisting of three factors and a total of 24 items. Subsequent CFA confirmed the validity of the dimensional structure we constructed. The research findings indicate that the scale we developed successfully captures all three dimensions theoretically, thus providing a comprehensive reflection of the interdisciplinary teaching readiness of the measured pre-service teachers.

Compared to previous studies that broadly examined teachers’ understanding of interdisciplinary teaching concepts and terminology [12], we first conducted a detailed subdivision of the concept of interdisciplinary teaching readiness. We decomposed it into three dimensions: interdisciplinary teaching knowledge structure readiness, interdisciplinary teaching skills readiness, and interdisciplinary teaching attitude readiness, thereby constructing a more systematic and operational survey framework. Secondly, we fully considered the particularity of pre-service teachers who have not yet participated in actual teaching. In the division of dimensions, we made a clear distinction between interdisciplinary teaching knowledge and interdisciplinary teaching skills. In the dimension of interdisciplinary teaching knowledge, we mainly drew on the Pedagogical Content Knowledge (PCK) theory to reflect the pre-service teachers’ readiness in interdisciplinary teaching knowledge accumulation. In the dimension of interdisciplinary teaching skills, we closely integrated actual teacher behaviors in teaching activities and subdivided it into various aspects such as interdisciplinary teaching design, implementation, and evaluation, thus better reflecting the emphasis on knowledge in pre-service teachers’ teaching readiness. In summary, the scale we designed has a clear structure and rich content, providing a comprehensive and scientifically valid measurement tool for assessing pre-service teachers’ interdisciplinary teaching readiness.

### Reliability and validity of the ITRS

The construct validity of ITRS was obtained through factor analysis. In principal component analysis, the three-factor structure of ITRS, accounting for 77.282% of the total variance, exceeded 60%, indicating that these three dimensions could effectively represent the entire data. In CFA, all items showed good standardized loadings on their respective latent constructs [77]. In reliability analysis, both the overall ITRS and the three factors had Cronbach’s α coefficients greater than 0.9, indicating internal consistency and stability within ITRS. The average variance extracted (AVE) for each factor indicated sufficient convergent validity for ITRS. Additionally, we established the discriminant validity of ITRS, demonstrating that these three factors measure different constructs of distinct phenomena.

### Theoretical and practical implications

Our study offers a novel framework and instrument for assessing the interdisciplinary teaching readiness of pre-service teachers. From a theoretical standpoint, the ITRS initially identifies three fundamental dimensions of interdisciplinary teaching readiness: interdisciplinary teaching knowledge structure readiness, interdisciplinary teaching skills readiness, interdisciplinary teaching attitudes readiness. This framework provides pre-service teachers and teacher educator with a comprehensive theoretical framework and guidelines on interdisciplinary teaching. It enables pre-service teachers to engage in self-improvement and assists teacher educators in developing a more nuanced understanding of the essential elements of interdisciplinary teaching preparation, thereby enabling them to make well-informed decisions regarding the content of training programs and to design more effective preparation programs [78,79]. Secondly, the ITRS developed in this study is one of a limited number of instruments designed to measure the readiness of pre-service teachers for interdisciplinary teaching in a Chinese university context. Despite China’s notable advancements in policy implementation and extensive coverage in promoting interdisciplinary teaching, a dearth of reliable instruments persists for comprehensively assessing pre-service teachers’ readiness for interdisciplinary teaching. Following rigorous factor analyses and reliability-validity tests, the ITRS was demonstrated to be a reliable and valid measurement instrument, thereby addressing a research gap in this area. It would be beneficial for future research to utilize the ITRS as a quantitative measure in order to gain insight into the readiness of pre-service teachers for interdisciplinary teaching.

From a practical standpoint, the ITRS developed in this study can facilitate the metacognitive enhancement of pre-service teachers, optimize the evaluation of the effectiveness of teacher education programmes, and inform the formulation of educational policies. The ITRS offers pre-service teachers the opportunity to identify their strengths and weaknesses in interdisciplinary teaching, thereby facilitating targeted self-reflection and improvement [80]. By analyzing the data provided by the ITRS, educational administrators can gain insight into the overall effectiveness and shortcomings of teacher education programmes. This enables them to make prompt adjustments to the programme content and training strategies, thus ensuring that the objectives of teacher preparation programmes are met. The ITRS offers valuable empirical data for policymakers. The results of the scale enable policy makers to develop more targeted policies and measures to ensure the effective implementation of teacher education programmes [81,82].

In conclusion, the ITRS developed in this study provides a reliable measurement instrument for pre-service teachers’ interdisciplinary teaching readiness. It offers a comprehensive understanding of interdisciplinary teaching readiness in theory and provides robust support in practice for pre-service teachers’ professional development, the evaluation of teacher education programmes, and the formulation of educational policies. The extensive utilisation of this instrument will facilitate enhancements to the general standard of teacher education in China, whilst also offering invaluable insights into the realm of pre-service teacher education globally.

## Conclusions

Interdisciplinary teaching is more crucial now than ever before. With an increasing number of primary and secondary schools transitioning to interdisciplinary learning, there is a growing demand for high-quality teachers who can thrive in this mode. Every student should have access to well-prepared teachers [83], making the demand for well-prepared teachers far surpass other aspects such as curriculum development. The framework for pre-service teacher interdisciplinary teaching readiness and the ITRS that we have created have demonstrated great potential for promoting the professional development of pre-service teachers, evaluating the effectiveness of teacher education programmes, and supporting education policy development.

Specifically, (1)The ITRS is a instrument designed to facilitate self-reflection and self-assessment among pre-service teachers [84], thereby enabling their professional growth [85]. The utilisation of the ITRS enabled pre-service teachers to gain a clear framework for assessing their readiness for interdisciplinary teaching as a way to think deeply about their strengths and weaknesses in their knowledge, attitudes, and skills. This comprehensive self-examination enables pre-service teachers to construct a personal development plan, thereby facilitating their adaptation to future educational roles and the acquisition of competence in interdisciplinary teaching.(2) The ITRS can be employed as a instrument for the assessment of the efficacy of teacher education programmes [86]. In addition to being utilized for self-assessment at the individual level, the ITRS can also be regarded as a reliable instrument for the evaluation of the overall effectiveness of teacher education programmes. Teacher educators may utilize the ITRS to ascertain information regarding the initial and final levels of interdisciplinary teaching readiness of pre-service teachers, thereby providing a more objective and accurate basis for programme evaluation. The analysis of pre- and post-test data enables teacher education institutions to identify deficiencies in their programmes in a timely manner, thus allowing them to implement the necessary improvements to ensure the quality and effectiveness of their teacher education programmes.(3) The ITRS functions as a support tool for the development of policy [87]. For those engaged in policy-making, the ITRS offers a scientific methodology for the collection and analysis of data pertaining to the readiness of pre-service teachers in China for the interdisciplinary teachings. This information can assist policy makers, education sector administrators, and heads of educational organizations in developing a more accurate understanding of the reality of pre-service teachers’ readiness for interdisciplinary teaching. This, in turn, enables them to formulate policies and measures that are aligned with national education development goals and that meet the needs of teachers’ personal growth.

Compared to previous frameworks and measurement questionnaires, the ITRS we developed is more concise and comprehensive, offering a broader impact and allowing for use by researchers and institutions alike. The ITRS not only serves to enhance the interdisciplinary teaching competence and professionalism of pre-service teachers, but also provides crucial data support and technical resources for the advancement of educational reform and development. We hope these results will pave the way for monitoring and cultivating interdisciplinary teaching among pre-service teachers.

## Limitations and future research

The results of this study must be viewed in the context of several limitations. Firstly, in the scale design, due to considerations of the difficulty and complexity of assessment tools, the ITRS only adopted Likert scale items. Although some items of the ITRS followed the principle of contextualized testing, overall, there were deficiencies in assessing certain key abilities. Future assessment tools could introduce more diversified question types, such as case analysis and essay questions, to more comprehensively reflect the comprehensive literacy of the respondents.

Secondly, the ITRS is subject to the limitation of self-reporting [88]. Although pre-service teachers know that survey results do not affect their grades, their response may be aimed at leaving a good impression on others or they may not be aware of whether they are adequately prepared. This could lead to biases beyond expectations in the results. While self-reporting plays a significant role in the domain of educational research, it is not a sufficient standalone measurement instrument [89]. The triangulation method (TM), which validates the same phenomenon in multiple ways, is particularly important for ensuring the comprehensiveness and accuracy of research [90,91]. Although the ITRS is effective in supporting self-reported data collection, it should ideally be supplemented with other forms of data in order to gain a deeper understanding of the research population. For example, in their study, Vriesema & McCaslin emphasise the value of self-report methods while also acknowledging their potential limitations [92]. They propose a multi-method strategy that integrates the investigation of self-reports with actual observations, thereby allowing for a broader understanding of students’ group dynamics. This approach not only acknowledges the significance of self-report data in elucidating the subjective experiences of individuals, but also enhances the reliability of the findings through external validation. Torrington et al. demonstrated that the relationship between student self-reports and teacher ratings pertaining to task performance exhibited a significant correlation, which provides further evidence of the validity of self-reports when used in conjunction with other measures [93]. In light of the aforementioned limitations, it is imperative to be fully aware of the boundaries of self-report applicability and to actively seek out supplementary measures such as classroom observations [94], trace data [95], teacher educator feedback [96], interviews [97], and other diverse data sources [89]. The adoption of a comprehensive assessment approach enables the researcher to gain a more objective and holistic understanding of the subject matter, while also effectively reducing the potential for bias that may result from relying solely on self-reports.

Finally, it is worth noting that the standards of the GFI and AGFI fit indices used in model comparisons during CFA are not optimal. For example, Jöreskog and Sörbom suggest that a GFI value greater than 0.90 is more widely adopted [98]. Nevertheless, in this study, we provided reasonable thresholds by considering the data volume and indicator conditions.

## Supporting information

S1 File Interdisciplinary teaching readiness scale. (DOCX)

S1 Data Sample A. (XLSX)

S2 Data Sample B. (XLSX)
