# Supplementary material for: Developing and validating an Interdisciplinary Teaching Readiness Scale (ITRS) for pre-service teachers in China
Source: PLoS One. 2024 Dec 31;19(12):e0315723. doi: 10.1371/journal.pone.0315723 (PMC11687813; doi:10.1371/journal.pone.0315723)
Supplement: S1 File — (DOCX) [file pone.0315723.s001.docx]

**Pre-service Teacher Interdisciplinary Teaching Readiness Scale**

Dear Pre-service Teachers,

Interdisciplinary teaching is a kind of problem-solving oriented teaching activity that integrates the knowledge and methods of two or more disciplines on the basis of focusing on the internal logic of each discipline.2022 The Compulsory Education Curriculum Program and Curriculum Standards issued by the Ministry of Education of China explicitly mentions that the teaching of various disciplines should devote no less than 10% of the class time to interdisciplinary teaching. class time for interdisciplinary theme teaching. As a teacher educator, preparation for interdisciplinary teaching is a matter of future career development.

This survey seeks to find out the real situation of your preparation for interdisciplinary teaching. The results of the survey will be used for research purposes only, and your responses are critical to us, so please be sure to answer carefully and in the context of your own personal reality. We promise that this survey will be conducted anonymously, and we will keep your personal information and answers strictly confidential, so please feel free to answer. Here to express my heartfelt thanks to you! Good luck in your studies! Instructions for completing the scale.

1. There is no right or wrong answer to the questionnaire, for each question, please tick the answer serial number that suits your own situation;

2. Please do not consult with others when filling out the questionnaire, do not omit questions, do not lose items.

**Basic Information**

1. Your gender

- Male
- Female
- Other

2. What is your university? (Please fill in the full name of the university)

3.What is your major?

- Humanities and Social Sciences
- Science and Engineering
- Others (please fill in the name of your school)

4. What is your grade level?

- Freshman
- Sophomore
- Junior
- Senior
- Other

5. What type of institution are you attending?

- Normal Universities and colleges
- Non normal Universities and colleges

6.What is your level of institutions?

- First-rate universities and disciplines
- Non first-rate universities and disciplines

7. What is the region of your institution? (According to the National Bureau of Statistics of China (NBS), the economic zones include: Eastern, Central, Western and Northeastern regions. Among them, the eastern 10 provinces (cities) including Beijing, Tianjin, Hebei, Shanghai, Jiangsu, Zhejiang, Fujian, Shandong, Guangdong and Hainan; central 6 provinces including Shanxi, Anhui, Jiangxi, Henan, Hubei and Hunan; western 12 provinces (districts, municipalities) including Inner Mongolia, Guangxi, Chongqing, Sichuan, Guizhou, Yunnan, Tibet, Shaanxi, Gansu, Qinghai, Ningxia and Xinjiang; northeastern 3 provinces including Liaoning, Jilin and Heilongjiang).

- Eastern
- Central
- Western
- Northeastern

8. Have you had any interdisciplinary or STEM learning experiences in elementary or secondary school?

- Yes
- No

9. Have you participated in any training or learning (e.g., courses, conferences, workshops, programs, etc.) related to “how to teach across disciplines”?

- Yes
- No

11. Have you ever had an interdisciplinary teaching or internship experience?

- Yes
- No

**Pre-service Teacher Interdisciplinary Teaching Readiness**

(Likert scale with five levels of “Strongly disagree = 1”, “Disagree = 2”, “Fairly agree = 4”, “Strongly agree = 5”). “Agree = 4”, ‘Strongly Agree = 5’.)

***Interdisciplinary teaching knowledge Structure***

(1) I am familiar with interdisciplinary teaching and related concepts (e.g., STEM education creator education, integrated practice activities, etc.)

- 1
- 2
- 3
- 4
- 5

(2) In April 2022, the Ministry of Education issued the Compulsory Education Curriculum Program and Curriculum Standards, which mentioned that each discipline should devote no less than 10% of class time to interdisciplinary thematic teaching, which is of great concern to me, and I understand the background and reasons for its emergence.

- 1
- 2
- 3
- 4
- 5

(3) In addition to mastering the knowledge of my own discipline, I also understand the knowledge system and content structure of other related disciplines.

- 1
- 2
- 3
- 4
- 5

(4) I am able to analyze the links between the knowledge of various disciplines according to the needs of interdisciplinary teaching and learning, following the logic of a problem or a project, and search for inter-disciplinary integration points.

- 1
- 2
- 3
- 4
- 5

(5) I am familiar with teaching methods such as problem-based learning, project-based learning, engineering design-based teaching, and the 5E teaching method.

- 1
- 2
- 3
- 4
- 5

(6) I am able to use appropriate instructional strategies to teach interdisciplinary content knowledge (e.g., engineering design-based learning activities to help students develop conceptual understanding of science)

- 1
- 2
- 3
- 4
- 5

***Interdisciplinary teaching skills***

(1) When designing cross-curricular teaching objectives, I am able to design appropriate and comprehensive objectives in close connection with the curriculum standards and by grade and subject.

- 1
- 2
- 3
- 4
- 5

(2) If I am asked to take on interdisciplinary teaching tasks, I can find appropriate learning themes of difficulty and complexity from students' real lives based on the content of the subjects.

- 1
- 2
- 3
- 4
- 5

(3) If I am tasked with cross-curricular teaching, I can design appropriate cross-curricular content based on specific teaching objectives and themes, taking into account different subject knowledge, social needs, and learners' experiences.

- 1
- 2
- 3
- 4
- 5

(4) If I am asked to take on interdisciplinary teaching tasks, I am able to choose appropriate interdisciplinary teaching models according to specific teaching conditions and requirements.

- 1
- 2
- 3
- 4
- 5

(5) If I am asked to undertake interdisciplinary teaching tasks, I can create interdisciplinary teaching situations according to teaching needs.

- 1
- 2
- 3
- 4
- 5

(6) If I am asked to undertake interdisciplinary teaching tasks, I am able to organize and carry out interdisciplinary teaching activities integrating two or more subjects.

- 1
- 2
- 3
- 4
- 5

(7) I am able to use teaching strategies, interdisciplinary collaboration, and multimedia technology to support interdisciplinary teaching and learning if I am asked to do so.

- 1
- 2
- 3
- 4
- 5

(8) If I am asked to take on interdisciplinary teaching tasks, I am able to guide students' inquiry, mobilize students' motivation, and stimulate students' interest.

- 1
- 2
- 3
- 4
- 5

(9) If I am asked to undertake interdisciplinary teaching tasks, I can organize students' self-assessment and peer assessment.

- 1
- 2
- 3
- 4
- 5

(10) If I am asked to take on interdisciplinary teaching tasks, I am able to assess students in multiple ways (e.g., collaborative interaction, critical thinking, application of knowledge, etc.).

- 1
- 2
- 3
- 4
- 5

(11) I am able to synthesize a variety of assessment methods to evaluate student learning if I am given the task of interdisciplinary teaching.

- 1
- 2
- 3
- 4
- 5

***Interdisciplinary teaching attitude***

(1) I love interdisciplinary education and recognize the significance and value of interdisciplinary education in the national talent strategy.

- 1
- 2
- 3
- 4
- 5

(2) I have a good understanding of interdisciplinary teaching.

- 1
- 2
- 3
- 4
- 5

(3) I would like to have the opportunity to participate in interdisciplinary teaching-related studies and training.

- 1
- 2
- 3
- 4
- 5

(4) I am willing to take the initiative to seek resources related to interdisciplinary teaching both inside and outside the university in order to improve my interdisciplinary teaching ability.

- 1
- 2
- 3
- 4
- 5

(5) I am willing to utilize data from research on teaching and learning to make timely adjustments to interdisciplinary instructional design and practice.

- 1
- 2
- 3
- 4
- 5

(6) I agree with the philosophy of interdisciplinary teaching and learning.

- 1
- 2
- 3
- 4
- 5

(7) I am interested in interdisciplinary teaching and learning.

- 1
- 2
- 3
- 4
- 5

(8) I am very much looking forward to interdisciplinary teaching in my future teaching work.

- 1
- 2
- 3
- 4
- 5
